# The isolation of early nuclear endosperm of *Oryza sativa* to facilitate gene expression analysis and screening imprinted genes

**DOI:** 10.1186/s13007-015-0092-4

**Published:** 2015-10-22

**Authors:** Quan Kuang, Xiaobo Yu, Xiongbo Peng, Meng-xiang Sun

**Affiliations:** College of Life Science, State Key Laboratory of Hybrid Rice, Wuhan University, Wuhan, 430072 China

**Keywords:** Rice, Endosperm, Parental-of-origin genes, Gene imprinting

## Abstract

**Background:**

Since the quality and yield of rice production depends on endosperm development, previous studies have focused on the molecular mechanism that regulates this developmental process. Recently, how this process is epigenetically regulated has become an important topic. However, the gene expression analysis and screening imprinted genes during early endosperm development remain challenging since the isolation of early endosperm has not been possible. Here, we report a procedure for the isolation of endosperm at 24 or 48 HAP (hours after pollination) during the free nuclear stage of endosperm development.

**Results:**

This technique allows for rapid and convenient collection of pure free nuclear endosperm. Early endosperm RNA can then be extracted from the isolated endosperm cells using dynabeads. Our results showed that the quality of RNA is satisfactory for gene expression analysis and screening the parental-of-origin specific genes in early endosperm.

**Conclusions:**

Thus, we offer a reliable method to overcome one of the major obstacles in the investigation of the molecular mechanisms of early endosperm development. Our approach can be used for accurate gene expression analysis and screening of imprinted genes, and facilitates the confirmation of endosperm-specific gene expression at the very early stages of endosperm development. This method could also be used in other species to collect early free nuclear endosperm.

## Background

The quality and yield of rice production depend strongly on endosperm development. Therefore, many studies have explored these developmental processes. Early studies focused mainly on morphology and structural observations. Whereas in last decade, more and more works dealt with molecular mechanisms regulating endosperm development.

The time course of endosperm development has been studied in several cultivars of rice. As reported in Purple Japonica rice and IR36, one sperm fusing with the central cell could be observed 4–5 h after flowering, forming the primary endosperm nucleus. About 6 h after flowering, the primary endosperm nucleus divides. About 24 h after flowering, the endosperm contains 8–16 free nuclei distributed around the periphery of the embryo sac [1, 2]. Gu et al. [3] also reported the time course of endosperm development in rice Yanjing 235. They found that 1 day after rice flowering, rice endosperm-free nuclei were mainly spherical. After 2 days, a rice embryo sac contained 1864 ± 211 free nuclei and the average mitotic cycle was 4.2 ± 0.07 h. After 3 days, rice endosperm cellularization occurred, after which rice endosperm cell number increased sharply. About 12 days after flowering, endosperm cell number peaked [3]. Later, Yang et al. [4] reported in more detail that rice primary endosperm nucleus divided first and produced two free nuclei at about 3.5 HAP (hours after pollination). The free nuclear number reached 10 at 6-7 HAP, and the number was about 50-80 at 1 DAP (day after pollination) [4]. Based on available data, Wang et al. [5] divided the entire rice endosperm developmental process into four stages: the free nuclear, cellularization, differentiation, and maturity periods. In general, rice endosperm development is of nuclear type. After the central cell fuses with a sperm the primary endosperm cell is created. Then, the nucleus of the primary endosperm cell divides several times without cell wall formation to form so called free nuclei. These nuclei are first peripherally located along the embryo sac wall and then entad distributed as the number of the nuclei are increased. The first 3 days after pollination represents the free nucleus period. During this period, the embryo sac is small and endosperm is syncytial, or there is no cell wall between the endosperm nuclei. In addition, the free nuclear endosperm is unevenly distributed throughout the embryo sac at early stages [5, 6]. Before free nuclei fully occupy the embryo sac, endosperm cellularization initiates at periphery rejoin and soon entad extends until the endosperm in the embryo sac is totally cellularized. Thus, the process of rice endosperm development has been well-described. However, the time course of endosperm development can vary among rice cultivars. In a recent study, it was shown that rice endosperm was in the free nuclear stage in seeds 2 days after fertilization (DAF) and became cellularized at 3 DAF [7]. Therefore, for gene expression analysis corresponding to specific developmental stages of endosperm development, the time course of endosperm development should be carefully investigated in specific cultivars.

The molecular mechanism of endosperm development remains unclear, and few studies have explored molecular regulation of endosperm at the free nuclear and cellularization stages due to technical limitations. RT-PCR is a sensitive method to test the gene expression in endosperm, unfortunately up to now it is no possible yet to analyze the gene expression patterns in early endosperm since collection of early endosperm cells are rather difficult. In addition, it has been reported that imprinted genes play an essential role in regulating this developmental process [8–13]. However, to date only 21 imprinted genes have been identified in flowering plants including eudicot *Arabidopsis* and monocot cereal rice and maize [14]. Some of PcG family genes have been mainly studied, such as *OsiEZ1*, *OsCLF*, *OsEMF2a*, *OsEMF2b*, *OsFIE1*, and *OsFIE2* [8, 9]. Among them, *OsFIE1* has been confirmed as a maternal expressed genes in endosperm. In addition, statistical analyses revealed that 262 candidate imprinted loci were in endosperm, and among them 56 loci were confirmed to be imprinted in rice seeds [7]. Further pioneering studies are required to obtain detailed information on gene imprinting in rice endosperm. Since the expression of imprinted genes may be developmental-stage–dependent, it is important to screen and confirm the imprinted genes in all stages of endosperm development.

To accurately analyze gene expression patterns and screen imprinted genes in endosperm, endosperm cells must be isolated. For mature or nearly mature endosperm cells, the isolation procedure is simple. Using manual dissection under a stereoscopic microscope, it is simple to obtain sufficient endosperm cells for observation or gene expression analysis since the embryo and endosperm are found in distinct compartments in rice seeds and they can be isolated as pure fractions. However, for early immature endosperm cells it remains difficult to isolate sufficient amounts of cells without contamination by other tissues. Luo et al. [9] presented a rice endosperm isolation method where they harvested hybrid endosperm by cutting a small hole in the top side of 20 young seeds and squeezing endosperm into a grinding pestle. This squeezing method can successfully isolate endosperm at 5 DAP, which is just after cellularization. The authors also noted that the method was not used for isolating endosperm at 4 DAP to reduce the possibility of maternal seed coat contamination, which may affect imprinting analysis of these non-endosperm-specific genes. At this time, successful isolation of the earliest endosperm for gene expression analysis is at 4**–**5 DAP in rice. In some early efforts, to study early endosperm the whole ovule after fertilization was used for RNA extraction and microarray analysis [15]. This is not suitable for screening imprinted genes due to the influence of maternal tissues.

Since 1–3 DAP is an essential period of endosperm development in rice, to understand the expression pattern of some critical genes and the behavior of imprinted genes in this period is essential to explore the molecular mechanism underlying endosperm development. Therefore, it is necessary to circumvent the technical limitations of early endosperm isolation. The primary difficulty of isolating endosperm at 1–3 DAP is that the endosperm are still in the free nuclear status and are not yet cellularized. Endosperm resembles a nuclear suspension in the embryo sac. It is difficult to separate the endosperm from other maternal tissues. By careful dissection in our pre-experiment, it is possible to obtain a few endosperm nuclei, but more time is required to collect sufficient material for analysis. At the same time, it is difficult to avoid the influence of released RNAases and various stresses during the isolation procedure.

To address this problem, we compared various techniques. Here, we report a reliable method that enables precise and efficient isolation of early rice endosperm at 24 and 48 HAP. A corresponding procedure of RNA extraction from these trace endosperm is also described. We show the feasibility of isolating endosperm cells using this technique. Therefore, we offer a reliable method to overcome one of the major obstacles in the investigation of molecular mechanisms of early endosperm development. Our approach enables screening of imprinted genes and facilitates the identification of endosperm-specific genes at the very early stages of endosperm development.

## Results

### Developmental process of early endosperm

**Fig. 1:**
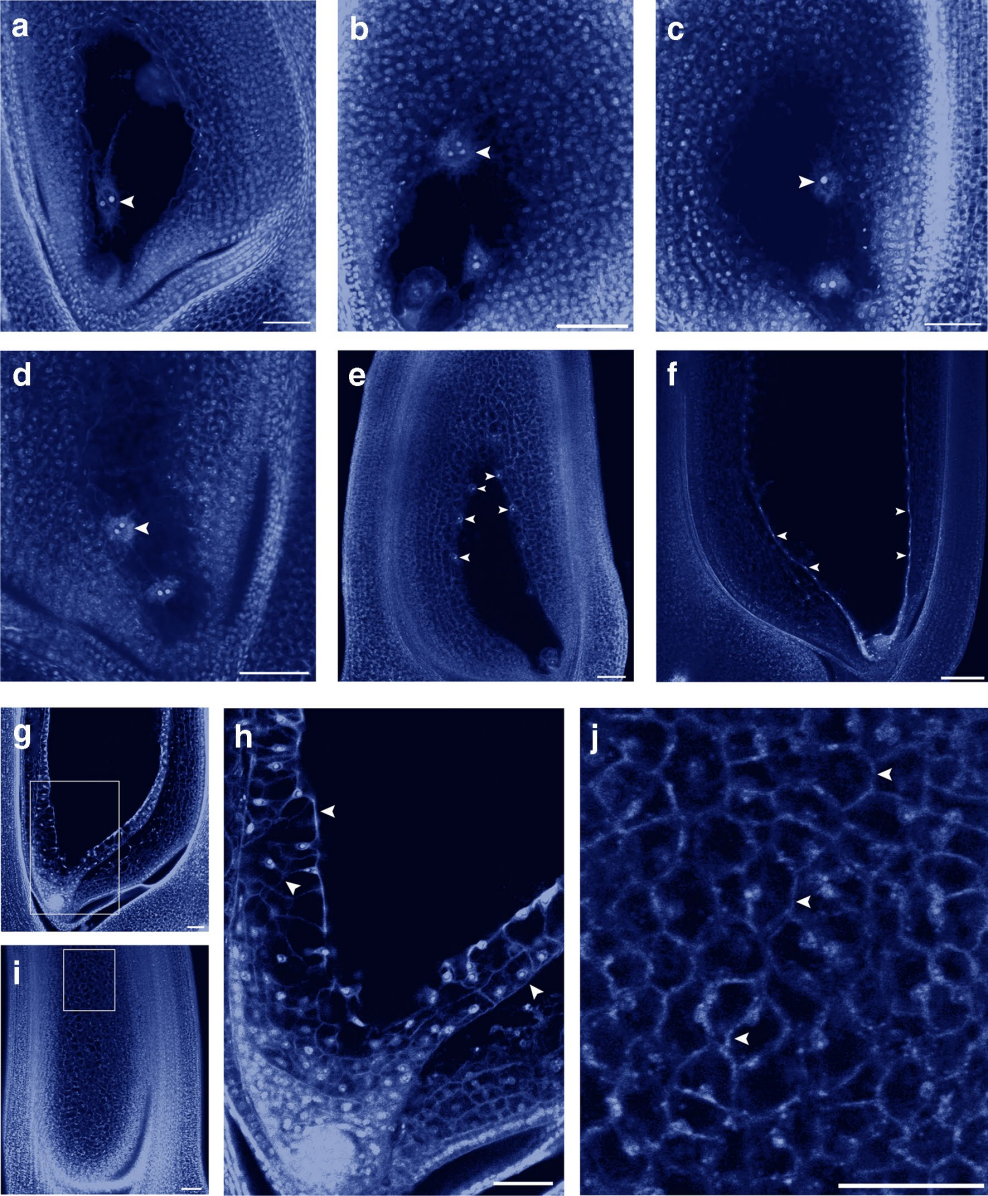
Early development of rice endosperm in hybrid of Nipponbare (♀) × 9311(♂). **a** Three hours after pollination (HAP), the central cell was not yet fertilized (*arrowhead*). **b** About 4 HAP, the central cell was fused with a sperm cell. The *arrowhead* indicates three nuclei in the central cell before nuclear fusion. **c** About 5 HAP, the three nuclei were fused and a primary endosperm nucleus was formed. **d** About 6 HAP, primary endosperm cells divided. The *arrowhead* indicates one of the two endosperm nuclei. **e** About 19 HAP, free endosperm nuclei (*arrowheads*) were distributed peripherally along the embryo sac. **f** About 46 HAP, free nuclei (*arrowheads*) constructed a complete network around the embryo sac. **g** About 67 HAP, endosperm cellularization was triggered. **h** Magnification of the *boxed region* in **g** to show cell wall formation (*arrowheads*). **i** About 96 HAP, the embryo sac was filled with endosperm nuclei. **j** Magnification of the *squared part* in **i** to show the cell wall (*arrows*). *Bar* 50 μm

Since the time course of endosperm development may vary according to cultivation conditions and cultivars, we carefully followed the developmental process of the hybrid between Nipponbare and 9311 under our conditions using propidium iodide (PI) staining combined with the confocal microscopy [16]. Our observations revealed that fertilization was not observed before 3 HAP (Fig. 1a). Later, double fertilization occurred and primary endosperm nucleus formed at 4-5 HAP (Fig. 1b, c). Around 6 HAP, the primary endosperm nucleus division was observed (Fig. 1d) and free nuclei were produced. At about 19 HAP, the endosperm nuclei appeared to be distributed peripherally around the embryo sac (Fig. 1e). About 46 HAP, the endosperm nuclei were fully distributed around the embryo sac periphery (Fig. 1f). The nuclei were positioned in the cytoplasmic matrix to form a network, in which each nucleus was separated almost equal-distantly from each other. About 67 HAP, cellularization processes were triggered from the outmost layer of endosperm, especially from the micropyle end around the embryo (Fig. 1g and h). At 96 HAP, the endosperm was obviously cellularized (Fig. 1i, j) and the embryo sac was full of endosperm cells. This time course confirmed that at 1-3 DAP the endosperm remained in its free nuclear status in the hybrid between Nipponbare and 9311. According to this developmental characteristic of endosperm, we designed a procedure for isolation of early endosperm at 1–3 DAP.

### Procedure for sample collection and observation of isolated endosperm nuclei

**Fig. 2:**
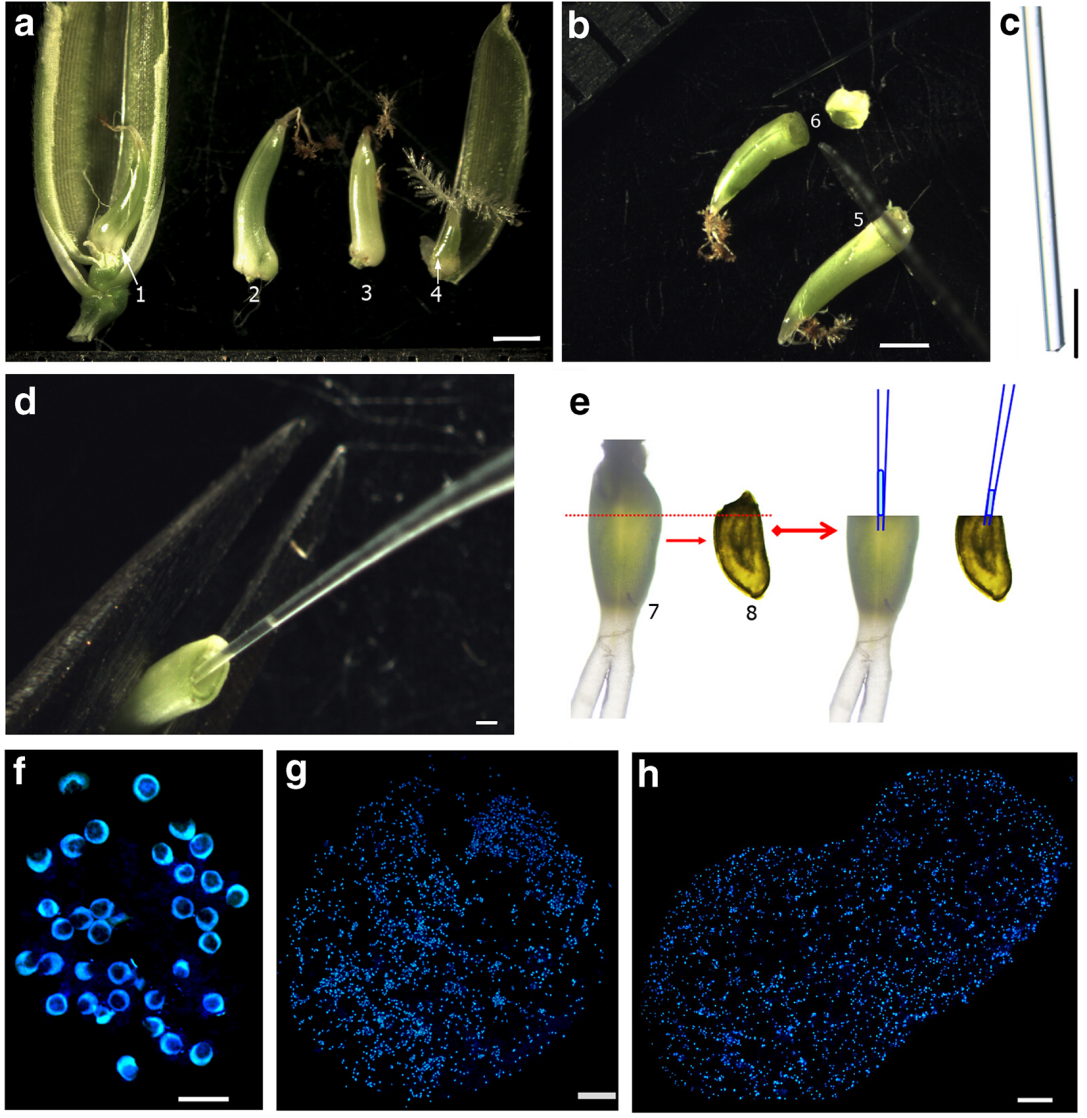
Isolation of nuclear endosperm. **a** Close-up view of ovaries. *1* The 9311 ovary at 24 HAP; *2* the 9311 ovary at 48 HAP; *3* nipponbare ovary at 48 HAP; *4* nipponbare ovary at 24 HAP. **b** An ovary was cut at the micropyle end (*5*) into two parts (*6*), with the cutting position shown. **c** The tip of a glass micropipette. **d** Sucking out nuclear endosperm using a micropipette. Note the volume of the sucked liquid-like endosperm can be clearly observed at the tip of the micropipette. **e** Schematic diagram of the process of cutting ovary and extracting endosperm. *7* Ovary; *8* an isolated ovule to show its position in relation to cutting line (*red line*). **f** The isolated endosperm free nuclei from an ovary of Nipponbare pollinated with 9311 pollen at 24 HAP, showing clear rice free nuclei without contamination by other cells. **g** The isolated endosperm free nuclei from an ovary of Nipponbare pollinated with 9311 pollen at 48 HAP. **h** The isolated endosperm free nuclei from two ovules of Nipponbare pollinated with 9311 pollen at 48 HAP. The nuclei in **f**–**h** were stained with DAPI. *Bar* 1 mm in **a**, **b**; *bar* 100 µm in **c**, **g** and **h**; *bar* 250 µm in **d**; *bar* 25 µm in **f**

The rice hybrid spikelets were collected at 24 and 48 HAP, respectively. Under a stereomicroscope, glumelle and lemma of hybrid spikelets were gently stripped with two tweezers (Fig. 2a) and only the ovaries were used for experiments. The handmade razor was used to cut the ovary rapidly at the top end and make a small hole in the embryo sac (Fig. 2b). The glass micropipette prepared in advance (Fig. 2c) was immediately inserted into the hole and was used to gently aspirate nuclear endosperm from the embryo sac (Fig. 2d). The collected drops of endosperm were directly released into 8 μl of lysis buffer. The lysis buffer containing endosperm could be directly used or temporarily stored in the refrigerator at −80 °C for extracting RNA at a convenient time. The manipulation procedure is illustrated in Fig. 2e, and the entire process can be completed in several seconds.

Using the methods described above, the ovaries could be cut and nuclear endosperm aspirated from them. To isolate pure endosperm without contamination by maternal tissues, we put the tip of the micropipette in the center of the embryo sac, ensuring that the micropipette tip did not come into contact with the embryo sac wall during the manipulation process to avoid wounding the embryo sac and absorbing cells from maternal integument tissues.

The isolated endosperm nuclei can be stained by DAPI and easily distinguished from the contaminated cells and maternal tissues since they are free nuclei without cell walls. As seen in Fig. 2f, bright blue fluorescence of free spherical endosperm nuclei was visualized using ultraviolet excitation from the fluorescence microscope. The clear background indicates the purity of extracted endosperm nuclei. Because there were fewer than 50 free nuclei per embryo sac at 24 HAP [4], to extract sufficient and quality RNA, endosperm must be collected from at least three embryo sacs. In contrast, when endosperm was collected at 48 HAP, only two ovaries were sufficient for the same purpose (Fig. 2g, h). In this case, to shorten the manipulation process, several ovules could be prepared previously, followed by continuous cutting and aspiration one ovule at a time. This process can be completed within a few minutes.

Since the endosperm could be collected rapidly and transferred to lysis buffer after separation from ovules, the possible influence of mechanical manipulation on gene expression can be minimized. Therefore, the pattern of gene expression in collected endosperm likely reflects the real pattern in vivo.

### RNA extraction from trace isolated endosperm

**Fig. 3:**
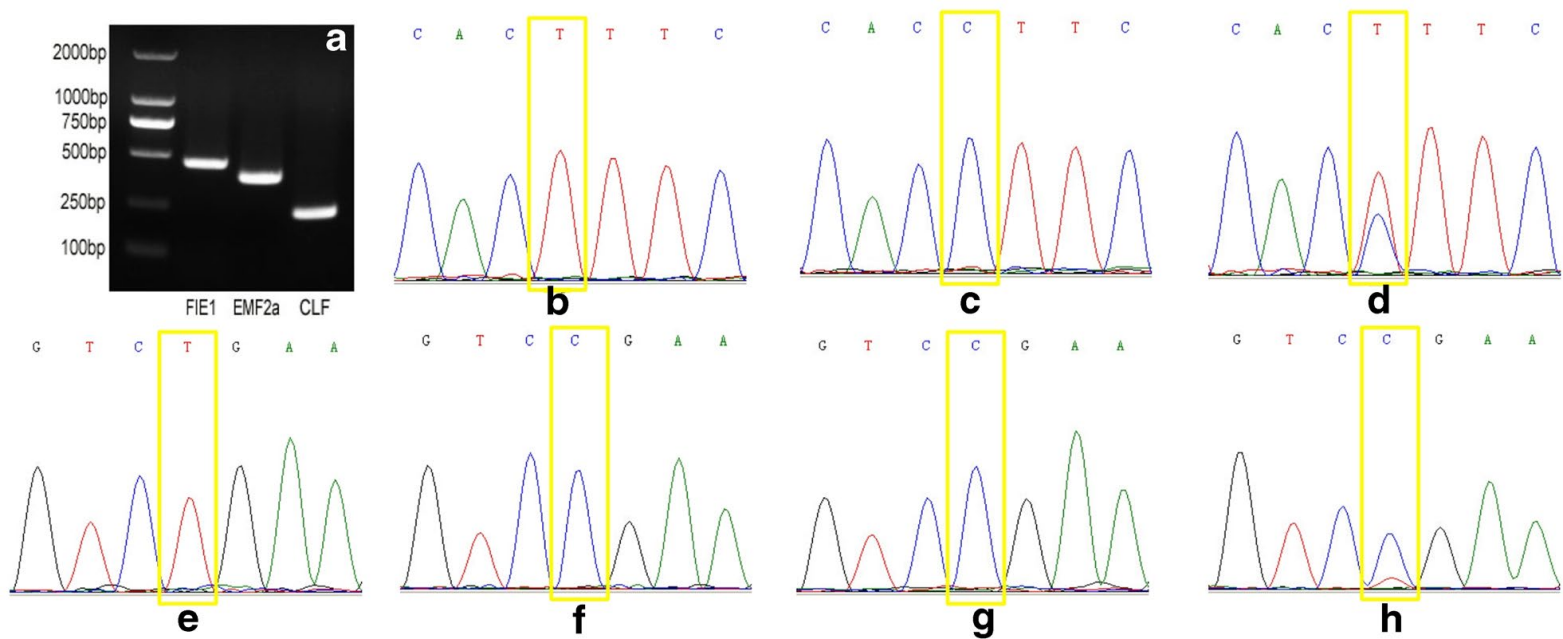
Quality analysis of RNA extracted from isolated hybrid endosperm. **a** RT-PCR electrophoresis bands of *OsFIE1, OsEMF2a* and *OsCLF.* **b** SNP of Nipponbare *OsCLF*. **c** SNP of 9311 *OsCLF*. **d** The SNP of “Nipponbare (♀) × 9311(♂)” heterozygote overlap-peak of *OsCLF*. **e** SNP of Nipponbare *OsEMF2a*; **f** SNP of 9311 *OsEMF2a*. **g** The SNP of “Nipponbare (♀) × 9311(♂)” heterozygote overlap-peak of *OsEMF2a*. **h** The contaminated overlap-peak of “Nipponbare (♀) ×9311(♂)” heterozygote OsEMF2a

Since the isolation process should be performed as soon as possible to avoid stress-induced gene expression, the amount of material and RNA isolated from the cells is limited. Generation of a quantity of PCR products sufficient to be visualized on agarose gels after amplification of target gene cDNA is necessary. Therefore, a procedure to minimize manipulation duration and to ensure production of sufficient cells to obtain reliable results is required. Endosperm cells collected from four ovules at 24 HAP within 5 min satisfy both the quantity and quality requirements. As shown in Fig. 3a, the 438 bp target fragment of *OsFIE1* was evident on the gel. This indicated that the endosperm isolated from these ovules is sufficient for gene expression analysis. In addition, since the primers were designed according to the sequence adjacent to the 5′ end, this result indicated that RNA degradation had been minimized. Otherwise, weak or smeared bands would have been present.

### Assessment of the quality of RNA for the analysis of gene expression

Theoretically, maternal and paternal alleles are equivalently expressed during seed development after fertilization. The endosperm is triploid and nuclei contain two maternal: one paternal genome complement resulting from the fusion of two central cell nuclei with a sperm cell nucleus. However, for imprinted gene expression, only maternal or paternal expression should be detected, otherwise there is contamination. Therefore, reliable methods of evaluating RNA quality are required. This method has been used to examine parental gene expression and is accepted as a useful tool [17].

We choose two genes, *OsCLF* and *OsEMF2a,* for testing. There are several tagged SNPs in the *OsCLF* gene of rice Nipponbare and 9311; for example, one tagged SNP is between primer C1 and C2: GAAGCCCTTGCTCAATCACT(C) TTCCAGGACCTGCCATGAG. The tag of Nipponbare is T (Fig. 3b) and the tag of 9311 is C (Fig. 3c). Based on our results (Fig. 3d), both maternal and paternal expression maintained an appropriate ratio, indicating that the quality of isolated endosperm cells was sufficient and there was no contamination with maternal tissue. Otherwise, if the isolated endosperm is contaminated with a mass of maternal tissues, such as nucellus cells from ovules, the maternal peak would be greatly increased, and thus the ratio would be markedly different.

In addition, there was one tagged SNP in the short transcript of *OsEMF2a* between primer H1 and H2: GTGCTCACAGCACATCTGGTCT(C)GAAGAC. The tag of Nipponbare was T (Fig. 3e) and the tag of 9311 was C (Fig. 3f). Since only paternal *OsEMF2a* short transcripts (Os04t0162100-02) (and no maternal *OsEMF2a* short transcripts) are present in early hybrid endosperm of “Nipponbare (♀) × 9311(♂)”, pure endosperm cells from hybrid ovules should show a clear single peak at the SNP site (Fig. 3g). On the contrary, if the sequencing peak figure shows not only a paternal peak but also a maternal peak (Fig. 3h), then some maternal nucellus cells are mixed with isolated endosperm and contributed to the maternal peak.

Using the methods described above, we confirmed that the isolated cells were sufficient for further analysis. Thus, as shown in Fig. 3, the isolated endosperm and RNA extracted from the limited amount of material could be used for assessment of parental gene expression.

**Fig. 4:**
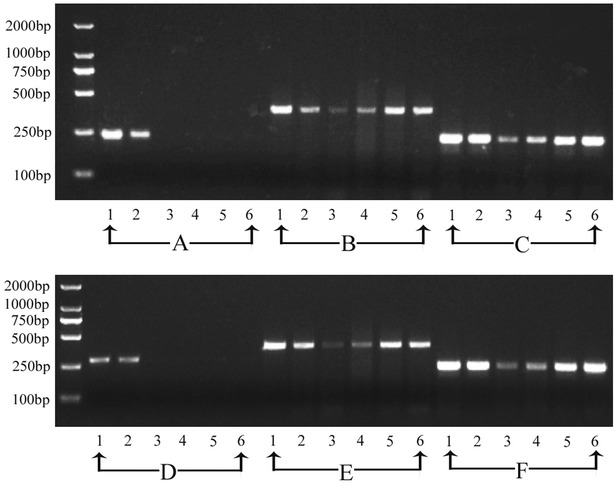
Confirmation of purity of isolated endosperm cells by RT-PCR. **a** *DX1* (Os06g21110); **b**, **e** *OsEMF2a* (Os04g08034); **c**, **f** *OsCLF* (Os06g16390); **d** *PsbR3* (Os08g10020); *1* leaf. *2* Ovule. *3-6* Endosperm isolated at the stages of 1dap, 2dap, 3dap, and 7dap, respectively

To further confirm the purity of the isolated endosperm we also selected other two genes, *DX1* (Os06g21110) [18] and *PsbR3* (Os08g10020) [19–22], which are universally expressed in ovule tissues and seed coat, but not in endosperm [18–22]. We tested the expression pattern of these genes by RT-PCR. As shown in Fig. 4, both genes are clearly expressed in leaf and ovule, but not in isolated endosperm at different developmental stages (the 1, 2, 3, 7 dap hybrid endosperm between Nipponbare and 9311). At the same time, two knowen genes in endosperm, *OsEMF2a* (Os04g08034) and *OsCLF* (Os06g16390), were well recognized in the isolated endosperm. This again demonstrates that the isolated endosperm was not contaminated with the maternal tissues and was of sufficient quality.

## Discussion

Gene imprinting in seed development has long been an important topic in the field of developmental biology. However, imprinted genes have been clearly identified only in endosperm cells; thus, endosperm development has become a major model for the study of gene imprinting and its role in specific developmental processes concerning the initiation of endosperm development, cellularization of endosperm, and seed yield. In fact, apart from the analysis of imprinted genes, the analysis of early endosperm-specific and developmental stage-specific genes is also critical for understanding regulatory mechanism of endosperm development and attractive to scientists in this field.

Previous studies have focused on the later stages of endosperm development due to technical limitations. Some pioneering studies have attempted to isolate early endosperm cells at 5 days after fertilization and reported isolation of sufficient endosperm cells for analysis [7, 8]. Overall, available techniques for the isolation of rice endosperm cells mainly include two approaches. One is manual dissection, which has been widely used for decades. Using this method, sections of endosperm at later developmental stages could be easily separated from the embryo and seed coat. Another method is the mechanical squeeze, which facilitates isolation of early endosperm cells by gently applying pressure on an ovule to release the delicate endosperm cells from the cut end [7]. These methods have been applied for the collection of mature and cellularized endosperm, respectively, but they are not suitable for isolation at the free nuclear stage because endosperm at this stage is a syncytium with suspended nuclei in an embryo sac. According to our results, it is relatively simple to dissect the ovules. However, identifying and collecting the nuclei using the dissection method is difficult. Using the squeeze method, it is possible to obtain some released nuclei, but neither can collect sufficient nuclei nor avoid contamination with other maternal tissues released by mechanical squeeze.

Considering the syncytium status of the early endosperm, we developed a simple and reliable method of isolating rice endosperm at 1–3 DAP. Thus, we provide a novel method with several unique characteristics: first, it is simple to perform. The entire process can be performed with little practice. In fact, only two steps need to be practiced: cutting the end of an ovule and aspirating the endosperm. Second, sufficient material can be collected within 5–10 min. If combined with the RNA isolation method shown in this work, there is no need to collect endosperm cells for analysis. Third, this approach ensures that isolated cells are fresh and original. A single transfer of endosperm from an ovule to lysis buffer can be completed within 5 s. This is especially important for gene expression analysis since it eliminates stress-induced gene expression and possible RNA degradation. Fourth, the contamination with maternal tissues is avoided. By gentle suction using a micropipette, only the syncytium endosperm is aspirated and collected, thus avoiding the release of other maternal cells from the ovules or seed coat together with endosperm nuclei, which can occur when the ovule is squeezed. As shown in this work, the purity of the isolated endosperm should be tested by RT-PCR amplification of specific genes prior to further analysis. This ensures the reliability of the material and thus also of the final results.

Concerning the application of the method, the following notes may be helpful.

1. Preparation of the micropipette. The easiest way to prepare the micropipette is by manually heating and pulling a commercial micropipette (e.g., Einmal-Mikropipetten, Germany) to produce a sharp end with a 25**–**30 μm opening. If not confident with manual preparation, any Micropipette Pullor (e.g. Narishinge PC-10, Japan) that is commonly used to prepare micropipettes for microinjection and patch clamp may be used. In addition, it is important to polish the cut end of the micropipette to ensure that the edge of the cut end mellow and smooth. A sharp opening of the micropipette can damage the embryo sac wall layer and increase the possibility of contamination with maternal tissues. Polishing of the sharp tip of the micropipette can be completed manually or using a Microforge (e.g. Narishinge MF830, Japan). Manual preparation is a convenient approach to polishing the cutting end. Typically, a rapid placing of the cutting end in the flame of a pocket-size alcohol burner is sufficient to render the end well-polished for use. The Microforge is also convenient since the procedure for manipulation is described in the instructions for the instrument.
2. Collection of endosperm cells. When aspirating the liquid-like free nuclear endosperm, the tip of the micropipette must be placed in the ovule and stopped at the center position, not the bottom of the embryo sac. In addition, collection of all endosperm cells in one embryo sac is inadvisable, but the majority of cells can be collected. This is also important for isolation of pure endosperm cells. It can be easily handled by a brief pre-experiment. First, aspirate as much of the contents of an embryo sac as possible and then mark a line on the tip of the micropipette. This line is visible under the stereo microscope. For collection, aspiration of ~70 % of this maximum volume is appropriate.
3. Evaluation of the purity of collected endosperm. After fertilization of the central cell, two female nuclei fused with one male nucleus with a theoretical maternal/paternal ratio of 2:1. Although the gene expression level may vary, under specific cultivation conditions and specific developmental stages the ratio is basically stable. Therefore, any maternal cell contamination may increase the maternal gene expression level; thus, whether the collected endosperm is contaminated with maternal tissues can be determined at an early stage in comparison with the control. However, the best marker is a paternally expressed gene in the endosperm. In this case, as described in this work, any maternal transcripts detected in the endosperm must have originated from contaminating maternal tissues.

## Conclusion

We developed a simple method for the isolation of rice endosperm as early as 1 DAP. The technique allows for the collection of pure endosperm and thus enables the screening of imprinted genes at the critical early stage. The method also facilitates confirmation of endosperm-specific gene expression by RT-PCR, which is typically evaluated by in situ hybridization or the expression of proteins fused with fluorescent markers. In addition, the technique should also be applicable for early endosperm isolation in other plant species.

## Methods

### Materials

*Oryza sativa* L. japonica. cv. Nipponbare and *Oryza sativa* L. ssp. Indica. 9311 were cultivated in the greenhouse of the state key laboratory of hybrid rice in Wuhan University with 13 h of illumination every day. The daytime temperature was 30 °C and the night temperature was 25 °C.

### Hybridization between rice cultivars

For testing the purity of isolated early rice endosperm and determining whether the isolated endosperm was contaminated, we isolated hybrid endosperm between two cultivars of rice; namely, Nipponbare and 9311. The Nipponbare spikelet was previously emasculated by removing immature anthers. The 9311 mature anthers were collected and the stigma of the emasculated Nipponbare spikelet was pollinated in sequence [23]. The reciprocal cross was also performed using the same approach.

### Preparation for the isolation of endosperm

Before the isolation of endosperm, razor blades, glass capillaries, and lysis buffer should be prepared for use.

1. Hand-made blades for cutting the ovary: commercially available razor blades were cut with sharp scissors into a 1 cm length, which was then fixed on a plastic stick for convenient handling.
2. Hand-made glass micropipette for endosperm isolation: The glass micropipette was prepared by heating and then manually pulling one end of the commercial glass capillary (1 mm in diameter) to form a tip with an opening of around 25**–**30 μm. The other end of the micropipette was connected to a sealed rubber hose.
3. Lysis buffer preparation: The lysis buffer was a mixture of 100 mM Tris–HCl, pH 7.5, 500 mM LiCl, 10 mM EDTA, pH 8.0, 1 % LiDS, and 5 mM dithiothreitol (DTT).

### DAPI staining of isolated early rice endosperm

According to the method described above, endosperm was collected under a stereomicroscope and then dripped into a droplet of DAPI (4′, 6-diamidino-2-phenylindole) dye liquor on glass slides. Observation was performed under an inverted fluorescence microscope (Olympus IMT-2) [16, 24, 25]. The images were collected using a cooled charge-coupled device (Cool SNAP HQ).

### Extraction of endosperm RNA

The endosperm RNA was extracted according to the Dynabeads^®^ mRNA DIRECT™ Micro Kit (Ambion/Life Technologies) [24–26]. The endosperm mRNA in lysis buffer was bonded with activated Dynabeads for about 5–20 min at room temperature with continuous rotation, and was then washed twice respectively with 100 μl of washing Buffer A, 100 μl of washing Buffer B, and 100 μl Tris–HCl in sequence. Finally, the Dynabeads-mRNA complex was resuspended in 8 μl reverse transcription PCR mix before cDNA synthesis.

### Reverse transcription of cDNA

The Superscript III reverse transcriptase system from Invitrogen Corp was adopted [26, 27]: 8 μl of reverse transcription PCR mix, including the Dynabeads-mRNA complex, were added to 1 µl of oligo (dT) and 1 µl dNTP mix. The mixture was heated to 65 °C for 5 min. Afterwards, 4 µl of 5X First-Strand Buffer, 1 µl DTT, 1 µl RNaseOUT™, and 1 µl SuperScript™ III were added and mixed by pipetting up and down gently before incubation at 50 °C for 60 min. Finally, the reaction was inactivated by heating at 70 °C for 15 min. The cDNA was then used as a template for amplification.

### Testing the quality of isolated rice endosperm and RNA

To determine the quality of isolated rice endosperm, we designed a pair of primers including a SNP of *OsCLF* (Os06g16390) (Table 1). Since both paternal and maternal alleles of *OsCLF* are expressed in early endosperm, they can be used to determine the ratio of parental expression of the gene. To further confirm the quality of the isolated endosperm cells, another pair of primers containing a SNP in the *OsEMF2a* (Os04g08034) short transcript (Os04t0162100-02) was deigned (Table 1). Only the paternal allele of *OsEMF2a* was expressed in early endosperm and was used to check for contamination with maternal tissues. Similarly, two seed coat expressed genes, *DX1* (Os06g21110) [18] and *PsbR3* (Os08g10020) [19–22] were also used for testing the purity of isolated endosperm. Pairs of primers for *DX1* (PM1 and PM2, The fragment size is 237 bp) and for *PsbR3* (PM3 and PM4, the fragment size is 286 bp) are listed in Table 2.

**Table 1:** Primers used in testing quality of isolated endosperm cells and RNA

Genes	Primers (5′–3′)
*OsCLF*	C1: GGAAAGAGGAATGTGTTGATGAGAG
	C2: CAAGAGAATCAACACCACTAAGAGC
*OsEMF2a*	H1: ACCTAGGCTGGAATACCGGATGTG
	H2: GTAAGCAAACGGGCCTGACTGAAC
*OsFIE1*	F11: CATCTCTCTTCCTCATCACCG
	F12: CTTCCCTCAGTGTGCTTGTTG

**Table 2:** Primers to test from depolluting

Genes	Primers (5′–3′)
*DX1*	PM1: AAGACCATGGTGGTGCTGTTCGTC
	PM2: TCATGGGTGAGAGTGGCCATAGC
*PD54O*	PM3: GAAGATCAAGACCGACAAGCCCTAC
	PM4: TCAGAAGCCCGGTGGTTCCTTTATG

To examine the quality of extracted RNA from the trace endosperm of rice, we selected a pair of primers of *OsFIE1* (Os08g04270) at the sequence next to the 5′ end to examine possible degradation (Table 1).
